# *FXN* protomutations are the source of pathogenic expanded GAA alleles in Friedreich ataxia and explain its unequal population distribution

**DOI:** 10.1093/hmg/ddag046

**Published:** 2026-06-09

**Authors:** Morgan C Devore, Christina Lam, Emily Xiao, Jeremy Devore, Kassandra R C McCain, Graham Wiley, Courtney C Park, David R Lynch, Sanjay I Bidichandani

**Affiliations:** Neuroscience Graduate Program, University of Oklahoma Health Sciences Center, 4000 N. Lincoln Blvd., Oklahoma City, OK 73104, United States; Department of Pediatrics, Section of Genetics, University of Oklahoma Health Sciences Center, 1200 Children's Ave., Oklahoma City, OK 73104, United States; Department of Pediatrics, Section of Genetics, University of Oklahoma Health Sciences Center, 1200 Children's Ave., Oklahoma City, OK 73104, United States; Department of Pediatrics, Section of Genetics, University of Oklahoma Health Sciences Center, 1200 Children's Ave., Oklahoma City, OK 73104, United States; Department of Pediatrics, Section of Genetics, University of Oklahoma Health Sciences Center, 1200 Children's Ave., Oklahoma City, OK 73104, United States; Clinical Genomics Center, Oklahoma Medical Research Foundation, 825 NE 13th St., Oklahoma City, OK 73104, United States; Division of Neurology, The Children’s Hospital of Philadelphia, 3615 Civic Center Blvd., Philadelphia, PA 19104, United States; Division of Neurology, The Children’s Hospital of Philadelphia, 3615 Civic Center Blvd., Philadelphia, PA 19104, United States; Neuroscience Graduate Program, University of Oklahoma Health Sciences Center, 4000 N. Lincoln Blvd., Oklahoma City, OK 73104, United States; Department of Pediatrics, Section of Genetics, University of Oklahoma Health Sciences Center, 1200 Children's Ave., Oklahoma City, OK 73104, United States

**Keywords:** Friedreich ataxia, repeat expansion, Protomutation, ancient DNA, population susceptibility

## Abstract

Friedreich ataxia (FRDA) is a recessive condition that is typically caused by inheriting an expanded GAA repeat (usually > 500 triplets) in the *FXN* gene from both parents who are heterozygous carriers of the expanded (E) allele. E alleles, which are evolutionarily derived from non-pathogenic long normal (LN) alleles (≥12 triplets), occasionally arise *de novo* via intergenerational expansion of premutation alleles (34–60 triplets). However, why FRDA susceptibility is limited to Eurasians, and how the prevalence of E alleles is sustained in susceptible populations are incompletely understood. Sequencing of the *FXN* locus revealed two major subclasses of E alleles, which have originated from a subset of Eurasian LN alleles, termed protomutations. Haplotype identity, the observed size continuum of protomutation-premutation-E alleles, and evidence of intergenerational instability in a protomutation allele, together support a dynamic relationship wherein protomutations can transition to premutation and E alleles. Consistent with the exclusive prevalence of FRDA in Eurasia, protomutations are absent in sub-Saharan Africa, where E alleles did not develop despite a relatively high prevalence of LN alleles. However, genetic admixture has introduced a slight risk of FRDA in African Americans. Analysis of ancient DNAs revealed that protomutations have existed in Europe and Western Asia for thousands of years, with evidence of spread to Europe via early Neolithic farmers. These data indicate that *FXN* protomutations serve as a reservoir for the generation of premutation and E alleles, and for millennia have sustained the geographically-defined population distribution of FRDA.

## Graphical Abstract

**Graphical Abstract:**
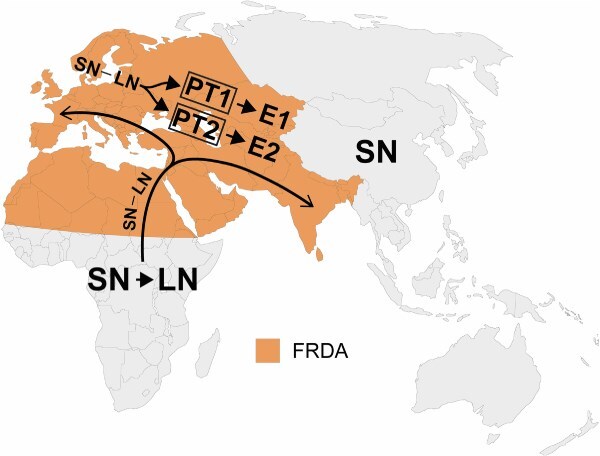

## Introduction

Friedreich ataxia (FRDA) is one of the most common inherited ataxias in people who trace their ancestry to Europe, West/Central/South Asia, and North Africa [1]. However, FRDA is almost never seen in people of sub-Saharan African and East Asian descent. FRDA is a recessive disease, and most patients inherit two copies of the *FXN* gene with an expanded GAA repeat (E allele) from parents who are both asymptomatic heterozygous carriers of the E allele [2]. The frequency of heterozygous carriers of E alleles is ~ 1% in susceptible populations [1, 3], thus the incidence of FRDA is maintained without the need for frequent *de novo* expansion events. In contrast, the absence of heterozygous carriers of E alleles makes FRDA exceedingly rare in non-susceptible populations [1]. The molecular basis for this difference in E allele prevalence in susceptible versus non-susceptible populations is not well understood.

The GAA repeat underlying FRDA is located at the center of an *Alu* element in intron 1 of the *FXN* gene [2], and thus exists only in primates. However, this GAA repeat has expanded specifically in humans, to become the E allele (with 56–1500 triplets; typically, > 500 triplets) that induces length-dependent epigenetic silencing of the *FXN* gene and causes FRDA [2, 4–9]. This expansion has occurred in a stepwise manner, initiated by a single ancient transition from short normal (SN; 5–11 triplets) to long normal (LN; 12–33 triplets) alleles, which occurred in Africa [1]. SN alleles constitute the most prevalent *FXN* allele, occurring at a frequency of 85%–90% in Europe and South Asia, 90% in sub-Saharan Africa, and > 99% in East Asia. LN alleles, which are absent in East Asia, make up most of the remainder alleles; i.e. 10% in sub-Saharan Africa and 10%–15% in Europe and South Asia [1, 3, 10]. Based on a shared haplotype between LN and E alleles, it is clear that all E alleles have originated from one or more LN alleles [1, 3, 11]. LN alleles are stably inherited [3, 10], and a direct transition from LN to E alleles has not been observed. However, rare alleles with 34–60 triplets, known as premutations, have been observed to expand directly into E alleles in a single (or few) intergenerational transmission(s) [3, 10]. How premutation and E alleles developed from LN alleles in Europe and South Asia, causing FRDA to develop there, and why it did not do so in sub-Saharan Africa, sparing that region from FRDA, remains unknown. We hypothesized that there must be an unrecognized step in the evolution of LN to premutation/E alleles that did not occur in sub-Saharan Africa, but which occurred exclusively in Eurasia, allowing FRDA to develop there.

Sequencing the entire *FXN* locus revealed that E alleles have arisen from a subset of longer LN alleles, which we term protomutation alleles. Protomutations have 14–37 pure GAA triplets, and are readily differentiated from LN alleles by their specific genetic signature, which is identical to that of E alleles. The allelic transition from LN to protomutation has occurred at least twice in human history, resulting in two major subclasses of E alleles. Consistent with the population distribution of FRDA, protomutations are seen only in susceptible populations, and are absent in sub-Saharan Africa. These data indicate that while the original SN to LN transition happened in Africa, at least two transitions from LN to protomutation alleles occurred relatively recently in Eurasia, thus explaining the geographically limited prevalence of FRDA. Protomutation alleles serve as a reservoir for the replenishment of E alleles, and are an indicator of population susceptibility to FRDA.

## Results

### Evidence of at least two major subclasses of E alleles in FRDA

**Figure 1:**
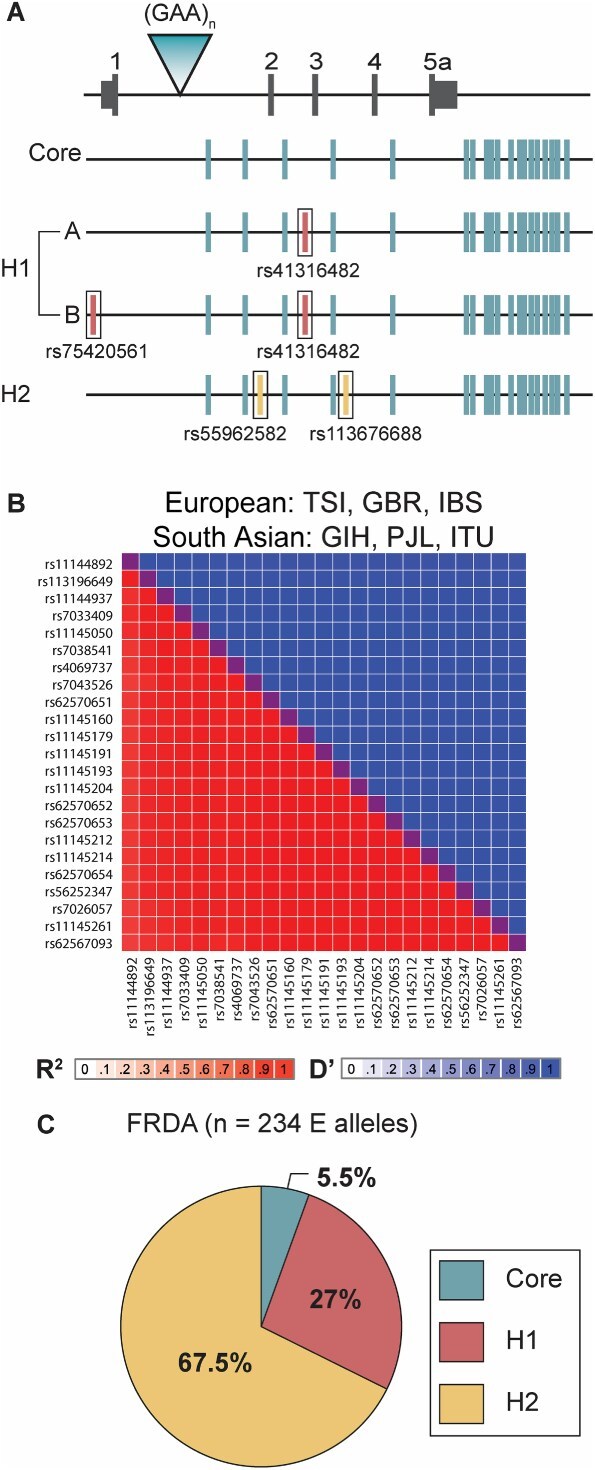
Two major subclasses of expanded (E) alleles in FRDA. (A) Schematic of the *FXN* locus showing the GAA repeat in intron 1 and exons 1-5a. Each horizontal line represents a distinct haplotype found in E alleles in FRDA, with vertical marks indicating SNPs spanning the *FXN* locus (drawn to scale). The 23 SNPs that define the FRDA core haplotype are shown in blue. The defining SNPs of the two major E allele haplotypes; i.e. H1 (subtypes a and B) and H2, are indicated in red and yellow, respectively (with SNP IDs indicated). (B) Pairwise linkage disequilibrium (LD) matrix for SNPs defining the core haplotype in Eurasian populations (i.e. European: TSI, GBR, IBS; and south Asian: GIH, PJL, ITU) from the 1000 genomes project. LD was calculated using the LDmatrix module of LDlink and is displayed as R^2^ (in red) and D′ (in blue). (C) Relative distribution of the major E alleles haplotypes seen in 234 E alleles from FRDA patients (mostly H1 or H2, with a minority carrying only the 23 SNP FRDA core haplotype, i.e. without additional defining variants).

To decipher the evolutionary origin of E alleles, the entire *FXN* genomic locus was sequenced in 15 FRDA patients (homozygous for E alleles), 3 heterozygous carriers of E alleles, and 3 non-FRDA controls; representing 33 E alleles [320–1260 triplets], 8 SN alleles, and 1 LN allele (Supplementary Data 1). In addition to targeted short-read genome sequencing of the entire *FXN* gene, several individuals were sequenced with whole genome longread sequencing in order to analyze the entire linkage disequilibrium (LD) block within which the *FXN* locus maps (Supplementary Data 1; Supplementary Fig. 1). A set of 23 SNPs was found to represent the core signature of E alleles (Fig. 1A), based on the following criteria: (i) presence in all E alleles; (ii) absence in SN alleles, and (iii) having a population frequency in Europe and South Asia of < 0.2, and in East Asia of < 0.05 (Supplementary Fig. 2A). The 23 core SNPs span the entire *FXN* gene on the 3′ side of the GAA repeat (Fig. 1A), and form a single core haplotype (*R*^2^ = 0.775–1.0 and D′ = 0.88–1.0) in European (TSI, GBR, IBS) and South Asian (GIH, PJL, ITU) populations (Fig. 1B; Supplementary Fig. 2B and C). Four transposon-related indels, within the LD block and spanning the *FXN* and *TJP2* genes, were detected in all (or most) E alleles by screening a set of FRDA patients (Supplementary Fig. 3A-D); however, because of their presence among SN alleles (Supplementary Fig. 3B) they did not meet the criteria for core markers of E alleles.

In addition to the core haplotype, E alleles showed one of two different sets of SNPs, thus representing two distinct haplotypes, H1 (A or B subtypes) and H2 (Fig. 1A). Genotyping of a total of 117 FRDA individuals (i.e. 234 E alleles; mostly of European ancestry) showed that the vast majority of E alleles have either the H1 (27%) or H2 (67.5%) haplotype (Fig. 1C). This indicates that there are two major subclasses of E alleles (i.e. E1 and E2 alleles), derived from two distinct originating events. However, despite their separate origins, E1 and E2 alleles are not materially different in terms of repeat length, or downstream consequences such as FRDA-DMR methylation, *FXN* transcriptional deficiency, and age of onset of symptoms (Supplementary Fig. 4A-D). While the majority of E alleles are on the H1 and H2 haplotype, as many as 5% of E alleles are not (Fig. 1C), indicating that E alleles have likely originated more than two times.

### E alleles have formed from protomutation alleles

**Figure 2:**
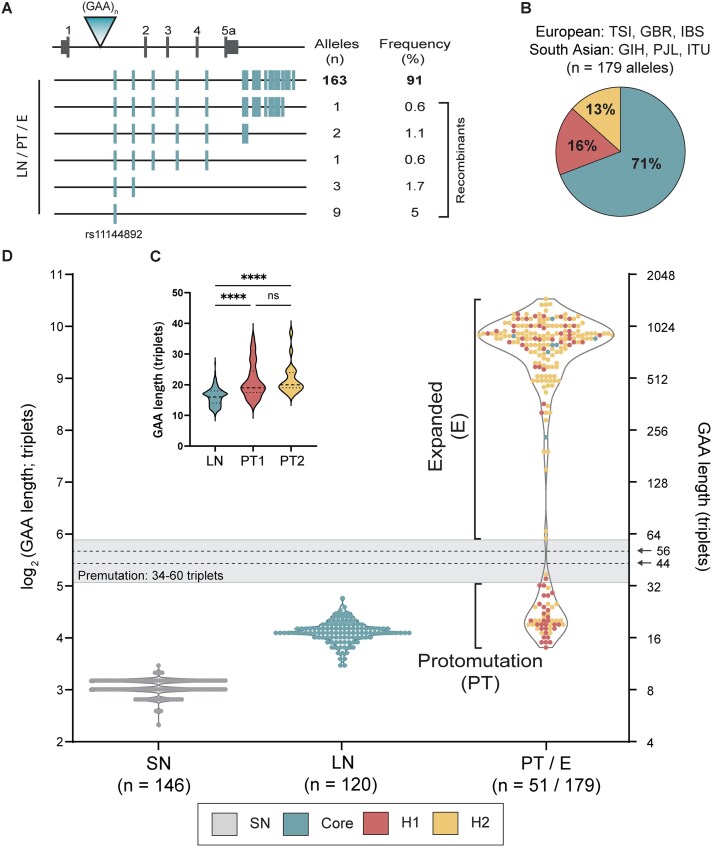
E alleles developed from a subset of LN alleles, called protomutation alleles. (A) the 23 SNP FRDA core haplotype, seen mostly in its entirety (91%), or as 3′ recombinants of the core haplotype, observed in LN and E alleles (*n* = 179 alleles) in Eurasian populations. Each horizontal line represents a distinct haplotype, with vertical marks indicating individual SNPs in the core haplotype. Allele counts (n) and frequencies (%) are indicated for all 179 alleles. (B) Distribution of the two major E allele haplotypes among individuals from Eurasian populations (TSI, GBR, IBS, GIH, PJL, ITU) who carry the core haplotype (*n* = 179 alleles). LN alleles with the two major haplotypes of E alleles (H1 or H2) are termed protomutations. **(C)** Comparison of GAA repeat lengths among non-disease alleles carrying the two major E allele haplotypes (H1 or H2), or only the core haplotype. Violin plots show the size distribution (in triplets), with medians indicated. ^****^P < 0.0001; ns = not significant (Mann–Whitney test). (D) Log₂-transformed GAA repeat length distribution for SN (*n* = 141), LN (*n* = 118), protomutation (*n* = 50), and E (*n* = 179) alleles. These data include individuals from six Eurasian populations (TSI, GBR, IBS, GIH, PJL, ITU) with the core haplotype, and 87 genotyped and informative FRDA patients. The left Y-axis shows log₂(GAA length in triplets), and the right Y-axis shows the corresponding GAA repeat length in triplets. The shaded box denotes the length threshold for premutation alleles (34–60 triplets), and brackets denote the distribution of protomutation and E alleles, below and above the premutation range, respectively. Note that while most E alleles are greater that the premutation size range, the shortest E alleles reported do map within this interval (dashed lines indicate these rare E alleles [56 and 44 triplets]).

To test the hypothesis that E alleles arose from a subset of LN alleles, we analyzed 604 individuals from six Eurasian populations in the 1000 Genomes Project [12] (IBS, GBR, TSI, GIH, ITU, PJL) for whom there was access to both genomic DNA and genomic sequence data. Genomic sequence was used to evaluate SNPs at the *FXN* locus. However, given the inadequacy of using available short-read genomic sequence data from the 1000 Genomes Project to precisely determine repeat lengths (see ‘Long-range PCR and repeat length determination’ in Methods), genomic DNA was used to amplify and sequence the GAA triplet-repeat in *FXN* intron 1. 179 alleles (in 161 [of 604] individuals) were found to have the FRDA core haplotype, with either the full set of 23 SNPs (91%), or partial haplotypes consistent with recombination events occurring 3′ of the GAA repeat (9%) (Fig. 2A). Long-range PCR plus longread amplicon sequencing detected repeat lengths consistent with LN alleles (*n* = 172) and E alleles (*n* = 5) [the remainder two alleles showed SN alleles, likely due to rare recombinants], reconfirming the common origin of Eurasian LN and E alleles. All individuals with E alleles were heterozygous carriers (66–1070 triplets; from TSI, ITU, PJL, GIH), representing a carrier frequency of 0.83%, which is close to what is expected for this Eurasian cohort [1, 3].

Of the 179 alleles with the core FRDA haplotype, 29% were also positive for the two haplotypes seen in the two major subclasses of E alleles [H1 (16%) and H2 (13%); Fig. 2B]. This indicates that both types of E alleles have originated from a subset of LN alleles, which we have termed protomutation alleles. Indeed, it is not possible to distinguish protomutations from E alleles simply by their genotypes (both have the same two major haplotypes). This also indicates that the H1 and H2 haplotypes were present at the time of (or shortly after) the two independent transitions from LN to protomutation alleles, and therefore must have preceded the formation of both subclasses of E alleles. When considered separately from LN alleles (which do not have the H1/H2 haplotypes), protomutation alleles contain 14–37 pure GAA repeats, and are significantly longer than LN alleles (Fig. 2C). While LN alleles rarely exceed 20 GAA triplets, protomutations frequently do (Fig. 2C; Supplementary Data 2). Thus, protomutation alleles differ from LN alleles in both haplotype and GAA repeat length, and constitute a distinct set of alleles (Supplementary Fig. 4A) that have so far been erroneously grouped with LN alleles. As expected, the two major haplotypes that define protomutations and E alleles are specific to modern humans, and are not seen in Neanderthal and Denisovan genomes, or in non-human primates (Supplementary Fig. 4B and C).

The precise determination of repeat lengths allowed detailed analysis of the size distribution of protomutations versus disease-causing and non-disease alleles. Sequencing of the 161 individuals described above resulted in accurate determination of repeat lengths in a range of alleles (note that most individuals were heterozygous for the core haplotype), including SN (*n* = 146), LN (*n* = 120), protomutation (*n* = 51), and E (*n* = 5) alleles from the six Eurasian populations. In order to increase the dataset of E alleles, long-range PCR plus rhAmp genotyping (rs11144892 for the core SNP set; rs41316482 for the H1 haplotype; rs113676688 for the H2 haplotype) was performed in a series of FRDA patients, which provided sizing and phased haplotypes for an additional 174 E alleles. As seen in Fig. 2D, both SN and LN alleles are below the size threshold of premutations (34–60 triplets), and form genotypically distinct allelic groups. Excluding the rare short E alleles reported to overlap with the premutation size range (56 and 44 triplets) [13, 14], most E alleles have > 100 triplets, and the majority have > 500 triplets (Fig. 2D). However, protomutation alleles, which are genotypically identical to both haplotypes of E alleles, constitute a defined set of alleles that bridges the size range from LN alleles to premutations/E alleles, thus resulting in a continuum from non-disease through to E alleles (Fig. 2D).

### Protomutation alleles may undergo small changes in repeat length via parental transmission

To test whether protomutation alleles are unstable via intergenerational transmission, we searched for parental transmission of protomutation alleles in all 192 Eurasian trios among 602 trios in the Expanded 1000 Genomes Project [15]. A total of 17 intergenerational transmissions involving protomutations were identified in 16 families (summarized in Supplementary Table 1); 8 from CEU, 8 from IBS, and 1 from PJL. Maternal (*n* = 11) and paternal (*n* = 6) transmissions of both PT1 (*n* = 10) and PT2 (*n* = 7) haplotypes were represented in this small cohort. Genomic DNAs were procured and long-range PCR followed by longread amplicon sequencing of all 17 parent–child combinations revealed a single change. A protomutation allele (PT2 haplotype) with 22 GAAs transitioned to one with 23 GAAs via paternal transmission (Supplementary Table 1). This indicates that protomutation alleles, i.e. those with GAA repeat lengths in the non-disease range, and below the size threshold for premutations, have the ability to mutate via intergenerational transmission, thereby producing small changes in repeat length.

### Lack of protomutation alleles explains the absence of FRDA in sub-Saharan Africa

**Figure 3:**
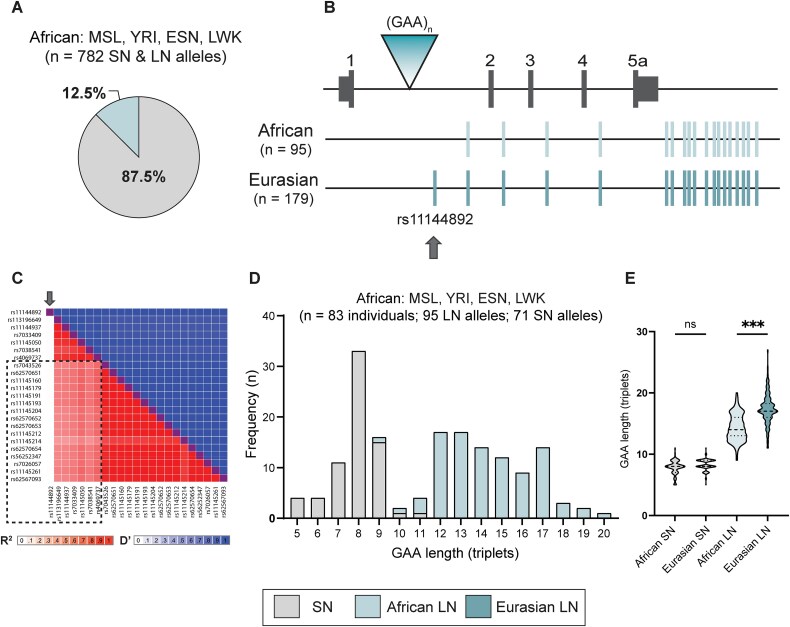
Absence of protomutation alleles in sub-Saharan Africa. (A) Distribution of alleles in sub-Saharan African populations (MSL, YRI, ESN, LWK; *n* = 782 alleles) showing only SN and LN alleles (grey indicates alleles lacking the core haplotype; blue indicates alleles carrying the African core haplotype). (B) Schematic of the *FXN* locus showing SNPs in the core-haplotype. Horizontal lines depict haplotypes in sub-Saharan Africans (*n* = 95 alleles; SNPs indicated in light blue) and Eurasians (*n* = 179 alleles; SNPs indicated in dark blue). The SNP closest to the GAA repeat (rs11144892), also in intron 1, which is characteristic of Eurasian LN alleles is absent in the African core haplotype (grey arrow). (C) Pairwise linkage disequilibrium (LD) matrix for SNPs defining the core haplotype in sub-Saharan African populations (MSL, YRI, ESN, LWK) from the 1000 genomes project. LD was calculated using the LDmatrix module of LDlink and is displayed as R^2^ (in red) and D′ (in blue), with color scales indicated. The vertical arrow notes the missing SNP (rs11144892; leftmost column) in the sub-Saharan African core haplotype. The dotted box highlights relative LD decay at the FXN locus. (D) GAA repeat length distribution (triplets) among all sub-Saharan African individuals (*n* = 83 individuals; MSL, YRI, ESN, LWK populations) found to carry at least one allele with the African core-haplotype, yielding 95 LN alleles (12 individuals were homozygous for LN alleles) and 71 SN alleles (71 individuals were heterozygous for LN alleles). (E) Comparison of GAA repeat lengths (triplets) for SN and LN alleles in sub-Saharan African versus Eurasian populations. The Eurasian set includes individuals carrying only the 23 SNP core haplotype, and excludes protomutation/E alleles. Violin plots show the size distribution, with medians indicated. ^***^P < 0.001; ns = not significant (Mann–Whitney test).

To investigate why E alleles and FRDA do not exist in sub-Saharan Africa, despite the presence of LN alleles, the *FXN* locus was analyzed in four sub-Saharan African populations from the 1000 Genomes Project (*n* = 391 individuals; MSL, YRI, ESN, and LWK). Approximately 12.5% of *FXN* alleles in sub-Saharan Africa had the core SNP haplotype indicative of LN alleles (Fig. 3A). Consistent with the absence of FRDA, the characteristic SNPs that are diagnostic of the two major haplotypes of protomutations and E alleles (i.e. H1 and H2) were not detected, indicating that protomutation and E alleles are absent in sub-Saharan Africa (i.e. all sub-Saharan *FXN* genes are either SN or LN alleles; Fig. 3A). This indicates that the absence of FRDA in sub-Saharan Africa is due to lack of the key transition from LN to protomutation alleles, which occurred subsequently and exclusively in Eurasia.

### Despite their greater evolutionary age, LN alleles in sub-Saharan Africa have not developed into *FXN* premutations

We next explored the possibility that given enough evolutionary time, LN alleles in sub-Saharan Africa could have transitioned to premutation alleles, i.e. in a parallel mechanism, independent of the Eurasian-specific transition to protomutation alleles. Instead of the 23 SNP core haplotype seen in Eurasian LN alleles, sub-Saharan African LN alleles were found to have a 22 SNP core haplotype, wherein the rs11144892 Eurasian core SNP is missing (Fig. 3B; Supplementary Fig. 6). Long-range PCR and longread amplicon sequencing of the GAA repeat in all available sub-Saharan alleles with the 22 SNP core haplotype (and derived 3′ recombinants [Supplementary Fig. 7A and B]; i.e. 83 individuals from MSL, YRI, ESN, and LWK) revealed LN alleles in all but one case (94 of 95 alleles; 99%; Fig. 3C). Conversely, analysis of the GAA repeat sequence in 155 alleles without the 22 SNP core haplotype (*n* = 88 MSL individuals) showed that all but one were SN alleles (154 of 155; 99%). This absolute association of LN alleles with the 22 SNP core haplotype indicates that a solitary transition from an SN to LN allele occurred in Africa, and preceded the acquisition of the rs11144892 SNP, which occurred later in Eurasia.

Evidence of LD decay (R*^2^* = 0.395–1.0; Fig. 3C [compare with Fig. 1B]), plus a relatively high recombination rate between SN and LN alleles (as many as ~ 13% of African SN alleles have acquired partial 3′ portions of the African core haplotype; Supplementary Fig. 7C), indicate that African LN alleles are evolutionarily much older than Eurasian LN alleles. Crucially, LN alleles in sub-Saharan Africa have not exceeded 20 GAA triplets (Fig. 3D), and have remained significantly shorter than their Eurasian counterparts (Fig. 3E; also note the slight overlap at the lower end of African LN alleles with SN alleles; Fig. 3D and E). This indicates that, despite the longer evolutionary time, African LN alleles have remained short and have not transitioned into premutation (and E) alleles. These data further support the hypothesis that transition to protomutation alleles, which occurred exclusively in Eurasia, was the key evolutionary step and a necessary prerequisite for the development of FRDA.

Interestingly, three LWK individuals were found to carry the full 23 SNP core ‘Eurasian haplotype’ (Supplementary Fig. 7). This is likely due to a low level of Eurasian genetic admixture, as is known for LWK [16]. Although, it is not possible to rule out the possibility that the ‘characteristic Eurasian’ rs11144892 SNP originally arose in Eastern Africa.

### Genetic admixture in African Americans has introduced protomutation and E alleles

**Figure 4:**
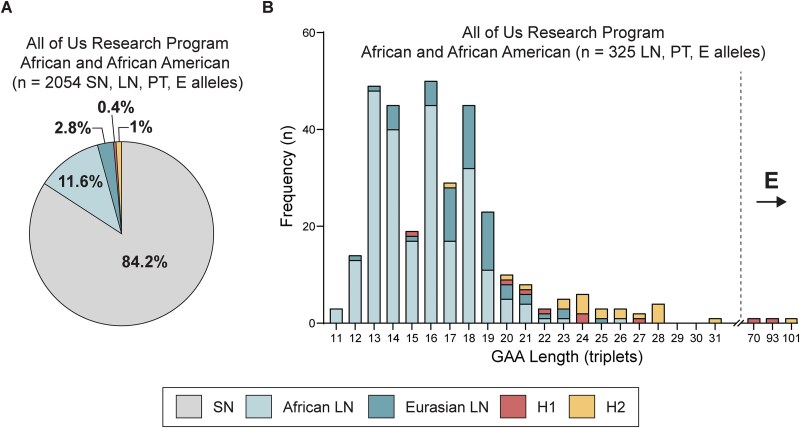
Genetic admixture in African Americans has introduced protomutation and E alleles. (A) Distribution of haplotypes among 2054 *FXN* alleles identified by long-read sequencing in 1027 African and African American individuals in the US (all of us research program). Relative proportions of the various alleles are shown based on their haplotype: SN alleles (lacking the core haplotype), LN alleles (carrying the African or Eurasian core haplotype), and protomutation/expanded alleles, carrying the two major haplotypes (H1 and H2). (B) GAA repeat length distribution (triplets) for all alleles carrying the African or Eurasian core haplotype (*n* = 325), showing the size and frequency of LN, PT, and E alleles. The dotted line indicates the threshold for disease-causing (E) alleles. Alleles carrying either of the two major haplotypes (H1 and H2) are defined as protomutation or E alleles if they are below or above this threshold, respectively. Alleles indicated by dark blue, red, and yellow colors are of Eurasian origin, and represent genetic admixture in this cohort.

The ability to clearly identify sub-Saharan LN alleles versus Eurasian LN and protomutation/E alleles based on haplotype allowed us to explore whether genetic admixture in African Americans has introduced a risk of developing FRDA. The All of Us Research Program [17]. which reported whole genome long-read sequences of 1027 African and African American individuals in the United States, allowed precise determination of both GAA repeat length and *FXN* haplotypes. Clear and quantitative evidence of Eurasian admixture was observed in this cohort. About 16% of the 2054 *FXN* alleles had haplotypes that were non-SN alleles (Fig. 4A), and slightly over a quarter of these (~27% [or 4.8% of all alleles]) were clearly non-African alleles marked by the 23 SNP core set. These included the entire range of Eurasian LN, protomutation, and E alleles. Consistent with *FXN* alleles in sub-Saharan Africa, the *bona fide* African LN alleles in this US-based cohort rarely surpassed 21 GAA triplets in length (Fig. 4B). Eurasian protomutation alleles (both H1 and H2 haplotypes) accounted for the majority of alleles exceeding 21 triplets in length. Three E alleles were identified in this cohort, representing an allele frequency of 0.1% in African Americans. This allelic distribution contrasts considerably with sub-Saharan African populations in the 1000 Genomes Project, and indicates that genetic admixture in African Americans has introduced a previously unrecognized, albeit slight, genetic susceptibility to FRDA.

### Variable prevalence of protomutation alleles across Eurasian populations

**Figure 5:**
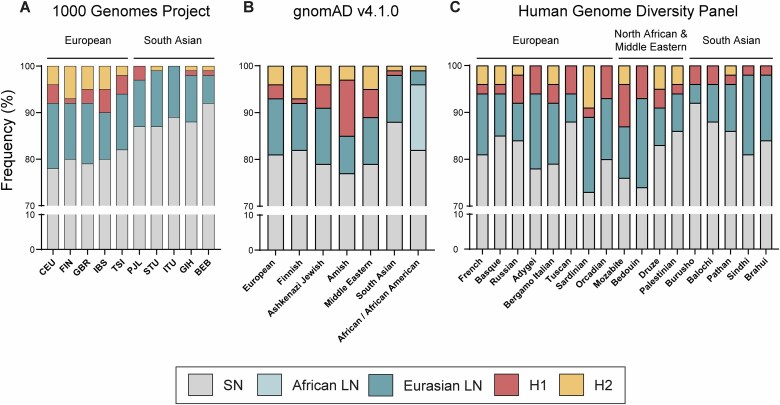
Variable prevalence of protomutation alleles across Eurasian populations. Stacked bar plots showing relative haplotype frequencies across populations from three datasets: (A) 1000 genomes project; (B) gnomAD v4.1.0; and (C) human genome diversity panel (HGDP). Each bar represents a single population/population group (labeled), and segments indicate the proportion of SN alleles, Eurasian LN alleles, African LN alleles, and each of the two major haplotypes of protomutation/E alleles (each allelic type is color-coded). Frequencies are depicted as a percentage of total alleles, within each population. Note: Only populations with one or both of the two major haplotypes of protomutation/E alleles are shown.

The prevalence of protomutation alleles was evaluated across diverse populations from various genomic repositories; including, the 1000 Genomes Project (*n* = 2017 individuals from 20 populations; note: Admixed American populations and non-Continental African populations were excluded), Genome Aggregation Database [18] (gnomAD; 7 populations), and the Human Genome Diversity Project [19] (*n* = 927 individuals from 54 populations). As expected, both LN and protomutation alleles were absent in East Asia, and protomutation allele frequencies in European and Middle Eastern populations were substantial (7%–10%; Fig. 5A-C; note: only populations with protomutation alleles are shown). In contrast, South Asians, despite being susceptible to FRDA, showed significantly lower frequencies of protomutation alleles compared with Europeans (e.g. 1.31% in STU, ITU, GIH, BEB, and PJL versus 8.18% in CEU, FIN, GBR, IBS, and TSI; Fisher’s exact test, *P* < 0.0001; see Fig. 5A [also see various South Asian populations in Fig. 5B and C]). Elevated frequencies of protomutation alleles were observed in several smaller populations, including the Amish, Israeli Druze, Ashkenazi Jews, Algerian Mozabites, and Sardinians. These data suggest that the incidence of FRDA in Eurasian populations is likely to be variable.

### Protomutation alleles have existed in Eurasia for at least 9000 years

**Figure 6:**
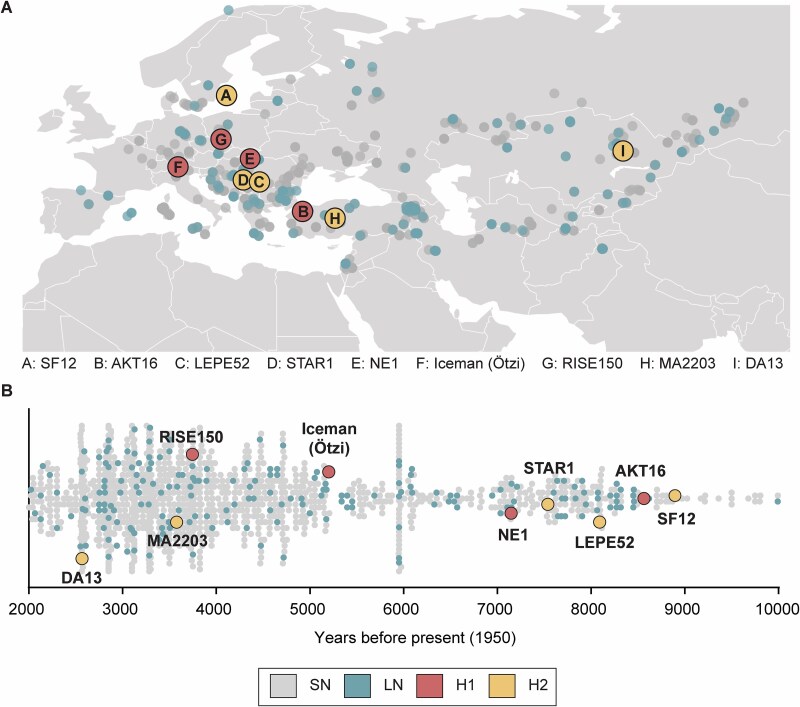
Protomutation alleles have existed in Eurasia for thousands of years. (A) Geographic distribution of ancient individuals carrying SN alleles, LN alleles, and either of the two major haplotypes of protomutation/E alleles. Each data point represents an ancient individual, plotted based on the geographic location of the human remains. (B) Temporal distribution of the same ancient individuals shown in panel a. each data point represents and ancient human individual plotted by age (years before present [i.e. 1950 ce]). Haplotype classifications are color-coded. Note: Only ancient individuals older that 2000 years before present are included in both plots.

Given the defined SNP signature, we explored the temporal and geographical distribution of protomutations in Eurasian ancient DNAs. Genetic data from ancient Eurasian individuals were sourced from various studies in which *FXN*-specific SNPs were available (Supplementary Table 2; Supplementary Data 3). The age range spanned 139–38 052 years before present (i.e. 1950 ce, by convention). SNPs in the core haplotype plus the two major haplotypes of Eurasian protomutations/E alleles were assessed in a total of 1848 informative ancient genomes (1286 that were > 2000 years old; 562 that were ≤ 2000 years old). SN and LN alleles were readily determined because the rs4069737 core SNP is included in the ‘1240 k’ array [20] (1.24 million informative SNPs spanning the human genome), which is widely used in ancient DNA studies. 1521 individuals (82.3%) had SN alleles, and 304 individuals (16.4%) had LN alleles (i.e. any Alt calls at one or more core SNPs), which is similar to the relative proportions of SN and LN alleles in current Eurasian populations. SN and LN alleles were similarly and widely distributed across Europe, and Western and Central Asia (Fig. 6A). Not surprisingly, LN alleles have existed in Eurasia for as long as SN alleles (at least 35 000 years in our cohort; Supplementary Data 3).

In contrast with SN and LN alleles, identification of protomutations was reliant on the chance sequencing of the respective diagnostic SNPs, which are not specifically enriched in ancient DNA studies. The H1 and H2 haplotypes of protomutation/E alleles were identified in 14 and 9 individuals, respectively. This is clearly an underestimate, since they represent only 7% of LN alleles in ancient DNAs versus 29% in current Eurasians. Both haplotypes of protomutation/E alleles have existed in Central and Eastern Europe as well as Western Asia (Fig. 6A) for at least 9000 years (Supplementary Table 2; Fig. 6B; only the 9 that were older than 2000 years are plotted). It is not possible to determine if these individuals have protomutations or E alleles [either zero or partial reads were noted at the *FXN* GAA repeat], although they are assumed to be protomutations given their much higher prevalence. Both haplotypes of protomutations are well represented among early Neolithic farmers in Anatolia (AKT16), and their descendants in Europe (STAR1, NE1, LEPE52; and Ötzi, who is a much later descendant), among ancient individuals prior to 7000 years before present (Supplementary Table 2). This is consistent with the arrival of protomutations in Europe as part of this seminal migration event in the early Neolithic period, which profoundly shaped the genetic makeup of Europe [21–23]. In more recent times (~1000 years ago), protomutations (especially the H1 haplotype) are well represented among Vikings (Supplementary Table 2) [24].

## Discussion

These data are consistent with a model of E allele evolution where a single, ancient, SN to LN allele transition occurred in Africa that was followed by at least two transitions from LN to protomutation alleles, exclusively in Eurasia. The genotypic identity of protomutations, premutations, and E alleles is consistent with the model that longer protomutations essentially assume the role of premutations, which are defined by their ability to transition directly to E alleles via intergenerational transmission. This model suggests that protomutations, which are below the size threshold of premutations, and have the ability to undergo small length changes via germline transmission, serve as the reservoir for the generation of E alleles, either directly or via premutations. Since the vast majority of FRDA patients inherit E alleles from heterozygous carrier patients [4], the need for replenishment of E alleles (which are lost at the population level via premature mortality in FRDA) is not great, and even very low frequencies of *de novo* E allele formation should suffice in order to maintain allelic equilibrium and stable FRDA incidence in susceptible populations. Since most E alleles have > 500 triplets, it is reasonable to assume that the majority of *de novo* E allele formation occurs via hyperexpansion of premutations [3, 10]. In this model, protomutations would serve as a steady source of premutations. In contrast, rare E alleles that contain < 250 triplets might arise via incremental transition from protomutations to E alleles, without hyperexpansion of premutations. Either way, protomutations are the ultimate source of E alleles, and serve to determine FRDA susceptibility in populations that carry these alleles.

The two LN to protomutation allele transitions likely occurred somewhere in Western Asia or in Europe, i.e. in the same geographical region where LN alleles migrated out of Africa. This geographically-specific generation of protomutations was the critical step that originally helped to establish FRDA susceptibility exclusively in Eurasia, and spared sub-Saharan Africa. Once protomutations were formed, the transition to E alleles likely occurred relatively quickly, based on the high linkage disequilibrium in both types of protomutation and E alleles (they have essentially the same two major haplotypes). Indeed, the fact that it occurred at least twice among Eurasians, with nearly identical outcomes in terms of the distribution of E allele sizes, indicates that the key step in developing FRDA susceptibility was to accumulate enough of a reservoir of alleles above a critical repeat length (i.e. protomutations).

Given the ability to identify protomutations from SNP data alone, and the trove of available ancient DNA data (which do not reliably report repeat sequences), we searched for direct evidence of the establishment and spread of FRDA susceptibility in prehistoric times. It is noteworthy that E alleles cannot be distinguished from protomutations by haplotype alone, so it is unclear when E alleles *per se* would have appeared (we took the conservative approach, and assumed that all instances of H1 and H2 haplotypes in the ancient DNA record represent protomutations because they are [and would have been] far more prevalent than E alleles). Furthermore, the detection of protomutation alleles is grossly underestimated in our study because the diagnostic SNPs of the H1 and H2 haplotypes are not enriched in typical ancient DNA sequencing libraries, and their detection is merely by happenstance. Despite these limitations, certain valuable clues were detected, which helped to broaden our understanding of the development of FRDA susceptibility.

Europe is known to have a gradient of FRDA prevalence; higher in the South and reducing in the north-easterly direction [25–27]. The molecular basis for this gradient is not clear. One study [26] that attempted to explain this gradient preceded our current nuanced understanding of the complex prehistoric migration events that have shaped the genetic makeup of Europe. Protomutation alleles in the ancient DNA record indicate that they were already present in Mesolithic European hunter-gatherers [28], consistent with the calculated age of the foundational mutation underlying FRDA [29]. However, protomutations (both H1 and H2 haplotypes) were found in several key ancient individuals representing early neolithic farmers originating in Anatolia, and their spread into Europe [21]. AKT16 represents the ‘source’ population of Anatolian neolithic farmers, indicating that protomutations were already present in Anatolia. Various key individuals representing early European farmers who descended from the source population in Anatolia and migrated into Europe (via the Balkans and Eastern Europe) also showed protomutations; STAR1 and NE1 represent neolithic farmers with minimal hunter-gatherer admixture, and LEPE52 represents a related population with considerable European hunter-gatherer admixture. Indeed, Ötzi (the ‘Iceman’), who is a much later descendant, and a relic (likely due to genetic isolation in the Alps) of neolithic farmers, with very high levels of Anatolian ancestry [30], exemplifies European persistence of protomutations acquired via neolithic farmer ancestry. These data are consistent with a wave of protomutation alleles (both H1 and H2 haplotypes) arriving in Eastern and Central Europe along the continental migratory route of early farmers from Anatolia. Later Bronze age migrations from the Steppe significantly modified the Neolithic farmer ancestry in Europe [31]. Interestingly, the residual gradient of Neolithic farmer ancestry in present-day Europe, which is highest in southern Europe and diminishes northwards [31], suggests a plausible role in determining the current gradient of FRDA prevalence in Europe.

There is no evidence to suggest that protomutations were substantially more (or less) prevalent in ancient populations than it is today. LN alleles, which are much more reliably ascertained, represent almost the same proportion of all *FXN* alleles in ancient DNAs [16.4%] as in contemporary susceptible populations. Therefore, as is the case today, a large population base would have been required for FRDA to manifest in any meaningful manner.

Population migration and genetic admixture have no doubt continued to influence the geographical landscape of FRDA. Interestingly, protomutations are well-represented in Vikings (Supplementary Table 2), highlighting how more modern migration and genetic admixture events may have redistributed protomutations and, likely, FRDA susceptibility widely. The introduction of protomutations and E alleles in African Americans via genetic admixture is a historically recent feature, and indicates a low level of FRDA susceptibility, which neurologists need to be cognizant of. This is similar to the introduction of FRDA susceptibility in Latin American mestizo populations via European admixture in native populations of the Americas [32].

The wide variability in the prevalence of protomutations in various Eurasian populations suggests that FRDA incidence is likely to be regionally variable, even in populations known to be susceptible to FRDA. For instance, it is striking how low the protomutation allele frequency is in South Asian populations relative to Europeans. While this may indicate a relatively low incidence rate, a very real weakness of our study is that one or more South Asian-specific protomutation alleles may have been missed due to the inadvertent underrepresentation of South Asians in the FRDA patient cohort (at the Children’s Hospital of Philadelphia) that was used to identify E allele-specific haplotypes. Any potentially missed South Asian-specific protomutations would therefore be indistinguishable from the general category of LN alleles, thus erroneously seeming like a low frequency of protomutation alleles in South Asia (when, in fact, it might simply mean that South Asians have a low frequency of *European* protomutation alleles). The ancient DNA record is also not helpful in this regard because of its inherent bias in favor of geographical regions that permit adequate preservation of human remains and DNA. The resulting underrepresentation of ancient DNA data in South Asia precludes the estimation of the time when protomutations, and therefore FRDA susceptibility, arrived in South Asia.

Collectively, these findings define a stepwise model for the evolutionary origin of FRDA susceptibility, and provide a framework for understanding its unequal global distribution.

## Materials and methods

### Study participants

Peripheral blood was obtained from individuals with a molecularly confirmed diagnosis of Friedreich ataxia who were enrolled at the Children’s Hospital of Philadelphia (CHOP). Fresh blood was collected in EDTA tubes and transported on ice via overnight courier to the University of Oklahoma Health Sciences Center (OUHSC) in Oklahoma City. All study procedures were conducted under protocols approved by the institutional review boards at both CHOP and OUHSC (CHOP IRB 25–023116; OUHSC IRBs 15 111 & 18 177), with written informed consent obtained from all participants.

Genomic DNA from the 1000 Genomes Project [12] was obtained from the Coriell Institute for Medical Research, including population panels representing three European populations: Iberian Population in Spain (IBS; *n* = 100 individuals), Toscani in Italy (TSI; *n* = 104 individuals), and British in England and Scotland (GBR; *n* = 99 individuals); three South Asian populations: Punjabi in Lahore, Pakistan (PJL; *n* = 108 individuals), Indian Telugu in the United Kingdom (ITU; *n* = 112 individuals), and Gujarati Indians in Houston, Texas (GIH; *n* = 109 individuals); and four sub-Saharan African populations: Mende in Sierra Leone (MSL; *n* = 98 individuals), Yoruba in Ibadan, Nigeria (YRI; *n* = 120 individuals), Esan in Nigeria (ESN; *n* = 111 individuals), and Luhya in Webuye, Kenya (LWK; *n* = 120 individuals). In addition, genomic DNAs from selected Eurasian parent–child duos (*n* = 16) representing 17 instances of transmission of a protomutation allele were obtained, from IBS, PJL and Utah Residents with Northern and Western European Ancestry (CEU) populations (Supplementary Table 1). Population abbreviations standardized by the 1000 Genomes Project [12] are used throughout the manuscript.

### DNA isolation

Genomic DNA used for PCR was extracted using the QIAamp DNA Blood Midi Kit (Qiagen; 51 185). Long-range PCR amplicons were purified prior to longread sequencing using the Wizard SV Gel and PCR Clean-Up System (Promega; A9282).

### HaloPlex targeted short-read sequencing of the *FXN* locus

HaloPlex HS libraries were prepared using the HaloPlex HS Target Enrichment System (Agilent) according to the manufacturer’s protocol. Briefly, 50 ng of genomic DNA per sample was digested using 16 restriction enzymes arranged as 8 double-digest reactions, with DNA split evenly across reactions. An enrichment control DNA (ECD) sample was processed in parallel. Completion of digestion was assessed using the ECD sample on a TapeStation system (Agilent) prior to downstream processing. Following digestion, DNA from all reactions per sample was pooled and hybridized to custom-designed biotinylated HaloPlex HS probes targeting the entire *FXN* locus, including 10 kb upstream and 10 kb downstream flanking regions. Hybridized DNA fragments were ligated to form circular molecules and captured using streptavidin-coated beads. Captured libraries were PCR-amplified, purified, and pooled for multiplexed sequencing. Sequencing was performed on the Illumina MiniSeq platform (Illumina, San Diego, CA) in standalone mode using paired-end reads (2 × 150 bp) with index reads of 8 cycles (i7) and 10 cycles (i5). Base call (BCL) files generated by the instrument were converted to FASTQ format using Illumina bcl2fastq software. Sequence data were analyzed using Agilent SureCall software to assess single-nucleotide and structural variants. The ECD control DNA was prepared and sequenced alongside each library to monitor digestion efficiency, library preparation quality, and sequencing performance.

### Genomic long-read sequencing

Oxford Nanopore longread whole-genome sequencing data analyzed in this study were generated previously and have been described in Devore et al (2026). Longread sequencing data were used to interrogate the full linkage disequilibrium block spanning the *FXN* locus (chr9:69007181–69 236 526; GRCh38), and to resolve genomic regions that were incompletely captured by HaloPlex short-read sequencing (Supplementary Data 1).

### Long-range PCR and repeat length determination

GAA repeat lengths were determined by long-range PCR, using AccuStart Long Range SuperMix (QuantaBio; 95 199–100) and T3F and T7R PCR primers, as described in Devore et al (2026) [33]. All FRDA patients, and individuals from the 1000 Genomes Project who carried the Eurasian or African core haplotype, and multiple parent–child duos from the Expanded 1000 Genomes Project [15], were analyzed. PCR amplicons were purified and subjected to longread deep sequencing (Oxford Nanopore Technologies); see note below. Amplicon libraries were prepared using Rapid Sequencing Kit V14 (Oxford Nanopore Technologies; SQK-RAD114) as per the manufacturer’s protocol, and as described in Devore et al. (2026) [33]. Repeat lengths were determined using trgt v1.2.0 (Tandem Repeat Genotyping Tool) and confirmed by manual inspection of individual sequencing reads. Manual curation was performed primarily for samples in which trgt-derived repeat lengths were discordant with haplotype-based expectations or with repeat size estimates inferred from agarose gel electrophoresis.

Note: Whole genome short-read sequencing data are available for individuals in the 1000 Genomes Project (and the Expanded 1000 Genomes Project). However, given our need to precisely determine GAA repeat lengths (in both *FXN* alleles), we explored whether resequencing with longread amplicon sequencing [33] would offer a superior option. A side-by-side comparison of both methods was performed in a test cohort composed of all IBS individuals who were known to be positive (i.e. at least one allele) for the FRDA core haplotype (*n* = 32 individuals; Supplementary Table 3). Longread sequencing of PCR amplicons yielded an average of 3190 reads through the GAA repeat, permitting confident determination of the consensus sequence of both *FXN* alleles (Supplementary Data 2). In contrast, short-read whole genome sequences showed an average of 40× depth at the GAA repeat (Supplementary Table 3), although often not sequencing through the entire repeat. ExpansionHunter called repeat lengths that differed from high-confidence reads via longread amplicon sequencing in ~ 60% of individuals (19/32), while HipSTR missed the correct genotype in ~ 72% of individuals (23/32), very often calling the repeat length in only the SN allele (~60%), even though a LN/PT allele was present (Supplementary Table 3). This level of inaccuracy was unacceptable for our purpose, which ultimately justified the additional expense, time, and effort involved in obtaining genomic DNAs and resequencing the GAA repeat via longread amplicon sequencing [33].

### SNP genotyping

RhAmp (RNase H-dependent amplification) endpoint SNP genotyping was performed according to the manufacturer’s protocol (Integrated DNA Technologies). Each 10 μL reaction contained 10 ng of genomic DNA, rhAmp genotyping assay, IDT rhAmp genotyping Master Mix and Reporter Mix. Assays for rs75420561, rs113676688, rs11144892, and rs4069737 were available as predesigned rhAmp genotyping assays; whereas, rs41316482 was genotyped using a custom-designed rhAmp genotyping assay. SNPs rs41316482 and rs75420561 were used to distinguish H1A and H1B, while rs113676688 was used to identify H2. SNPs rs11144892 and rs4069737 were included to confirm the presence of the core haplotype and to assess recombination in FRDA patients. Amplification and endpoint genotyping were performed on a LightCycler 96 instrument (Roche) using VIC and FAM fluorophores, and genotypes were assigned based on allele-specific fluorescence signals.

### Assay of transposon-related indels at the *FXN* locus

PCR assays were designed to call the four indels detected within the LD block in which the *FXN* locus maps as shown in Supplementary Fig. 3. PCR cycling conditions were as follows: Initial denaturation at 94°C for 3 minutes followed by 30 cycles of 94°C for 1 minute, annealing at 58°C for 1 minute, and extension at 72°C for 2 minute for indel I, or extension at 72°C for 1 minute for indels II, IIIa, IIIb, and IV, followed by a final extension at 72°C for 10 minutes.

### Analysis of publicly available genomic data

#### 1000 Genomes project

Allele frequencies for all SNPs of interest were obtained from the 1000 Genomes Project dataset via the dbSNP database. Public variant data from the 1000 Genomes Project were obtained via the International Genome Sample Resource data portal. Chromosome 9 variant call format (VCF) files were restricted to the *FXN* locus using the Ensembl Data Slicing tool. Linkage disequilibrium (LD) structure across the *FXN* region was assessed using LDblockshow with VCFs from TSI, GBR, IBS, GIH, PJL and ITU. LD was evaluated across a 500 kb window upstream and downstream of the *FXN* GAA repeat. SNPs corresponding to the core, H1, and H2 haplotypes were extracted using VCFtools for identification of LN, PT and E alleles based on Eurasian and African haplotype structures.

#### All of Us research program

Long-read sequencing data from 1027 African and African American participants in the All of Us Research Program [17] were accessed through the All of Us Researcher Workbench. *FXN* haplotypes were assessed using surrogate SNPs: rs11144892 represented Eurasian LN alleles; rs113196649 (with the absence of rs11144892) represented sub-Saharan African LN alleles; rs75420561 and rs41316482 represented protomutation/E alleles 1A and 1B; and rs55962582 and rs113676688 were used to identify protomutation/E allele 2. Individual BAM files were downloaded into the controlled workspace, reads spanning the *FXN* GAA repeat were extracted, and GAA repeat lengths were determined using trgt v1.2.0.

#### Human Genome Diversity Project

A combined VCF containing all participants from the Human Genome Diversity Project was accessed from previously published whole genome variant data. VCFtools was used to extract SNPs defining the core haplotype and protomutation haplotypes. The presence of the core haplotype was used to classify LN alleles, H1 and H2 haplotypes were used to identify PT1 and PT2 alleles, and the absence of these haplotypes was used to classify short-normal (SN) alleles.

#### GnomAD

Population allele frequencies were queried from GnomAD v4.1.0 using the same surrogate SNP framework as for the All of Us Research Program.

#### Ancient DNAs

BAM files from previously published ancient DNA samples were downloaded. Reads overlapping the *FXN* locus were extracted using SAMtools, and joint genotyping was performed using GATK to determine genotypes at SNPs defining the core, H1 and H2 haplotypes. Accordingly, the presence of any core SNP was used as a surrogate for LN alleles, and the presence of any H1 or H2 SNP was used as a surrogate for protomutation alleles. The absence of core SNPs, combined with reference calls at these positions, was used to infer SN alleles. We reasoned that even if recombination placed a core, H1 or H2 SNP onto an SN allele, a close relative of that individual would have carried the corresponding LN or protomutation allele. Note: Due to the low depth and coverage that is typical of ancient DNA datasets, genotypes are often effectively haploid, with only a single allele typically read and reported at any given position (i.e. pseudo-haploid). Thus, all results reported here reflect a single observed allele per site. This further underrepresents LN and PT alleles, as there is an inherent ~ 50% probability of observing the alternate allele at any given SNP in heterozygous individuals (most people with an LN or PT allele are heterozygous, with the other locus having an SN allele). Samples with sparse coverage across core-SNP positions would further minimize the possibility of identifying LN and PT alleles.

#### Archaic human genomes and non-human primates

Haplotype structure across non-human primates and archaic human genomes was assessed using the UCSC Genome Browser (GRCh37/hg19 assembly). Comparative genomics tracks were used for analysis of sequence conservation and haplotypic structure across the *FXN* locus. For archaic human genomes (Neanderthal and Denisovan), haplotypes were examined using the corresponding UCSC Genome Browser tracks derived from high-coverage ancient genome datasets. These tracks were used to visually identify sequence variation at positions corresponding to those in the core, H1, and H2 haplotypes.

### Statistical analyses

Statistical analyses were performed using GraphPad Prism v10.4.2. Linkage disequilibrium (LD) metrics, including R^2^ and D′ values, were calculated using the LDmatrix module of LDlink. The Mann–Whitney test was used to compare medians and interquartile ranges for non-normally distributed data.

### Data sharing

Longread sequencing data (BAM files) analyzed in this study were previously generated and are available in the National Center for Biotechnology Information (NCBI) Sequence Read Archive (SRA) under BioProject accession number PRJNA1304994 (Note: per IRB protocol, only sequence reads overlapping the *FXN* locus [chr9:69035752–69 079 076 in GRCh38] are made public.). Newly generated short-read genome sequencing data (FASTQ files) of the *FXN* locus are deposited in the NCBI-SRA under BioProject accession number PRJNA1454086. Consensus sequences derived by amplicon longread sequencing for all LN, PT, and E alleles identified in the 1000 Genomes Project (*n* = 244) are provided in Supplementary Data 2. *FXN* haplotype data (SN/LN/PT alleles) are provided for ancient DNA samples (*n* = 1848) in Supplementary Data 3.

## Supplementary Material

Supplementary_materials_ddag046
