# Supplementary material for: FXN protomutations are the source of pathogenic expanded GAA alleles in Friedreich ataxia and explain its unequal population distribution
Source: Hum Mol Genet. 2026 Jun 9;35(11):ddag046. doi: 10.1093/hmg/ddag046 (PMC13248553; doi:10.1093/hmg/ddag046)
Supplement: Supplementary_materials_ddag046 [file supplementary_materials_ddag046.zip › Devore et al Supplementary Materials Final.pdf]

Supplementary Materials

This file contains:

Supplementary Figures 1 - 7

Supplementary Tables 1 - 3

Description of Supplementary Data 1 - 3

Supplementary Figures

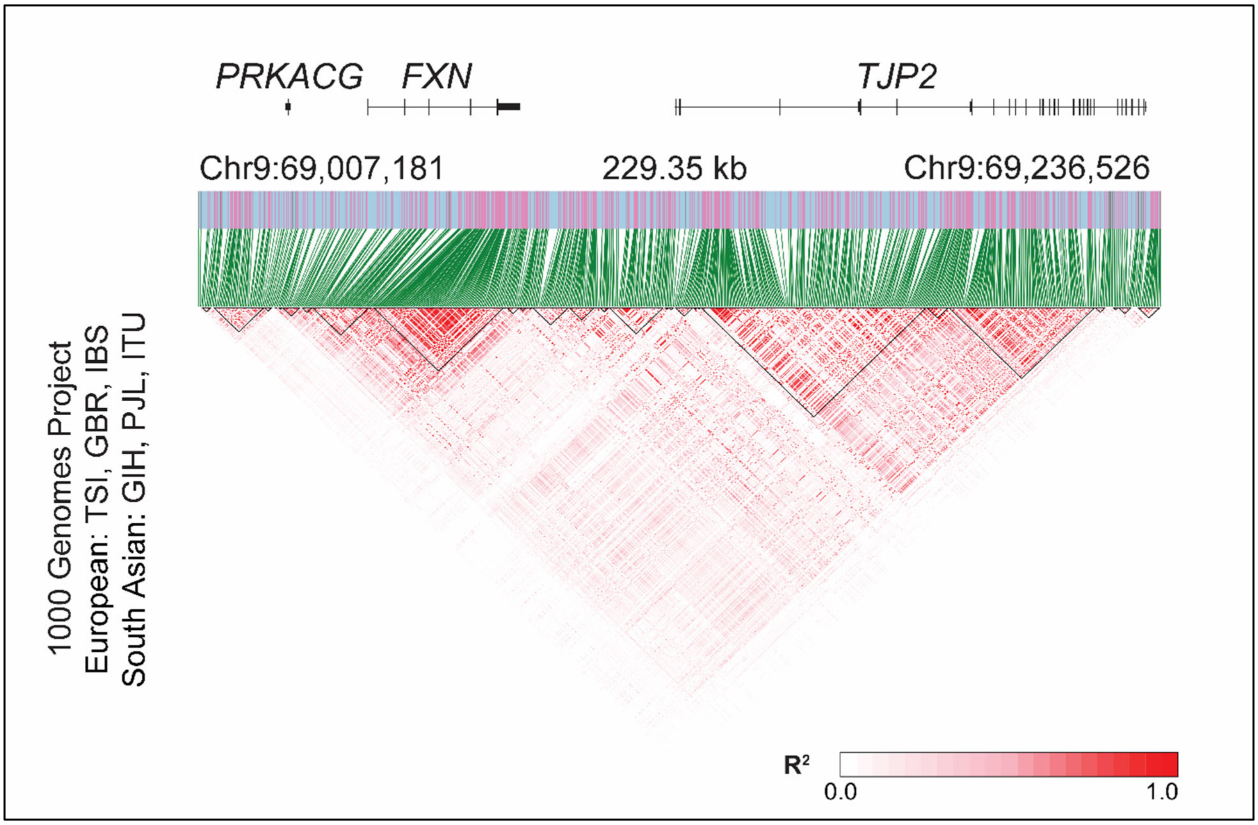

Supplementary Figure 1. Linkage disequilibrium (LD) block at the *FXN* locus. (A) Schematic of the genomic region on chromosome 9 spanning *PRKACG*, *FXN*, and *TJP2*. **(B)** LD pattern(s) visualized using LDblockshow for a ~229 kb region (Chr9:69,007,181 – 69,236,526; GRCh38), which corresponds to the major LD block identified within an initial ±500 kb search window centered on the *FXN* GAA repeat. LD is shown for Eurasian populations (European: TSI, GBR, IBS; and South Asian: GIH, PJL, ITU) from the 1000 Genomes Project. Pairwise LD is displayed as R^2 values, with color intensity values indicated in the scale bar.

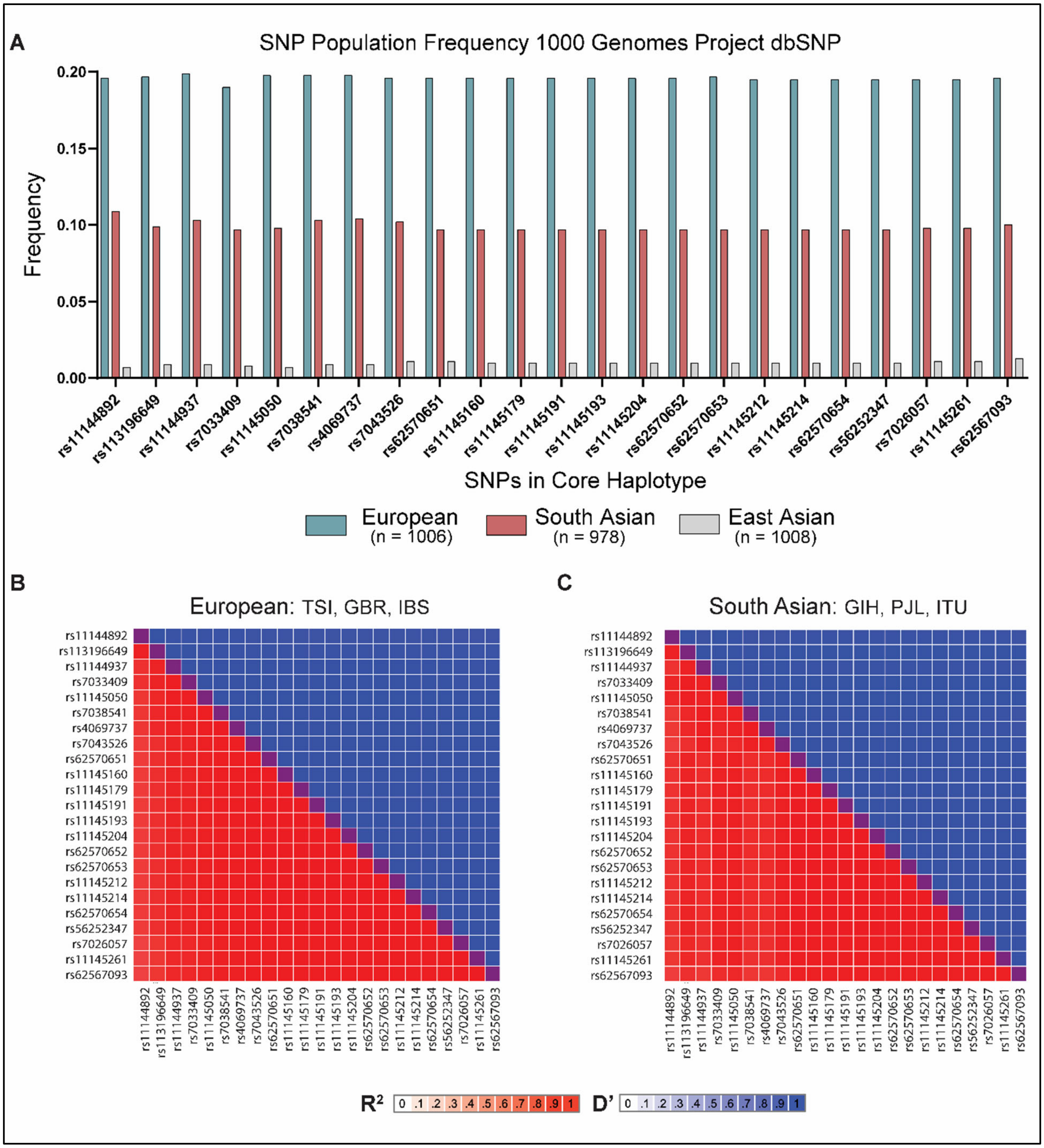

Supplementary Figure 2. Eurasian allele frequencies and linkage disequilibrium of SNPs in the FRDA core haplotype. **(A)** Allele frequencies of SNPs included in the FRDA core-haplotype (dbSNP; 1000 Genomes Project) in European, South Asian, and East Asian populations. **(B, C)** Pairwise linkage disequilibrium (LD) matrices for SNPs in the core haplotype in **(B)** European (TSI, GBR, IBS), and **(C)** South Asian (GIH, PJL, ITU) populations, generated using the LDmatrix module of LDlink. LD is shown using R^2 (red) and D' (blue), with color scales indicated.

A

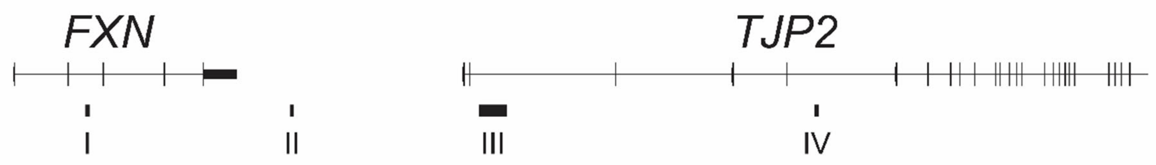

B

ID	Deletion Coordinants (GRCh38)	Size (bp)	Repetitive element	E alleles	SN alleles
I	chr9:69,050,407-69,050,641	234	*Alu*Y insertion	54 / 54 (100%)	24 / 24 (100%)
II	chr9:69,088,230-69,088,549	320	*Alu*Y insertion	54 / 54 (100%)	26 / 64 (41%)
III	chr9:69,123,197-69,128,441	5234	Likely L1 non-homologous recombination	54 / 54 (100%)	44 / 64 (69%)
IV	chr9:69,189,603-69,190,078	474	FAM/SINE – *Alu*Y non-homologous recombination	47 / 54 (87%)	23 / 64 (36%)

C

ID	Forward Primer (5'-3')	Reverse Primer (5'-3')	Size (bp) with deletion	Size (bp) without deletion
I	TCCTCACAGGGGCACAAAAG	TGGGGGAAAGTCCTTCACTG	1849	2086
II	ACCCTCAAGGACCCCTCAT	ATCTGGAAGAAGCAGGACTCA	606	934
IIIa	AGTGTTCACGTGTCTTGCCA	CAGAGGCCTAAGCCGCAATA	806	6040
IIIb	TTACCCAGTGCTCAACCTGC	GTTCTGCCAAATCTTGCCGT	NA	930
IV	TCCCCAGGACCCAGAATTGA	GCACCCACAACTCAAAGCAAA	802	325

D

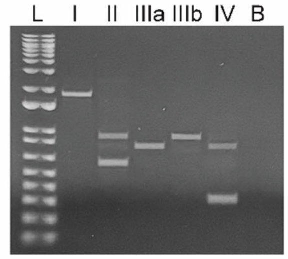

Supplementary Figure 3. Identification of insertions / deletions (indels) at the *FXN* locus by longread genomic sequencing. (A) Schematic of the *FXN*–*TJP2* genomic region (hg38) showing the positions of structural variants identified by longread genomic sequencing in FRDA patients. Deletions are labeled I through IV and are shown relative to the two genes. **(B)** Table summarizing the four identified indels, including genomic coordinates (GRCh38), size (bp), associated transposon / repetitive elements, and relative frequencies observed in E and SN alleles. **(C)** PCR primer sequences and expected amplicon sizes for each indel. PCR product sizes are indicated for alleles with and without the deletion. For deletion III, primer sets IIIa and IIIb were designed such that amplification occurs only when the deletion is present (IIIa) or absent (IIIb). **(D)** Representative agarose gel electrophoresis of amplicons showing deletions I–IV, with lane labels corresponding to primer sets shown in panel C. L = 1 kb ladder; B = zero template (blank) control.

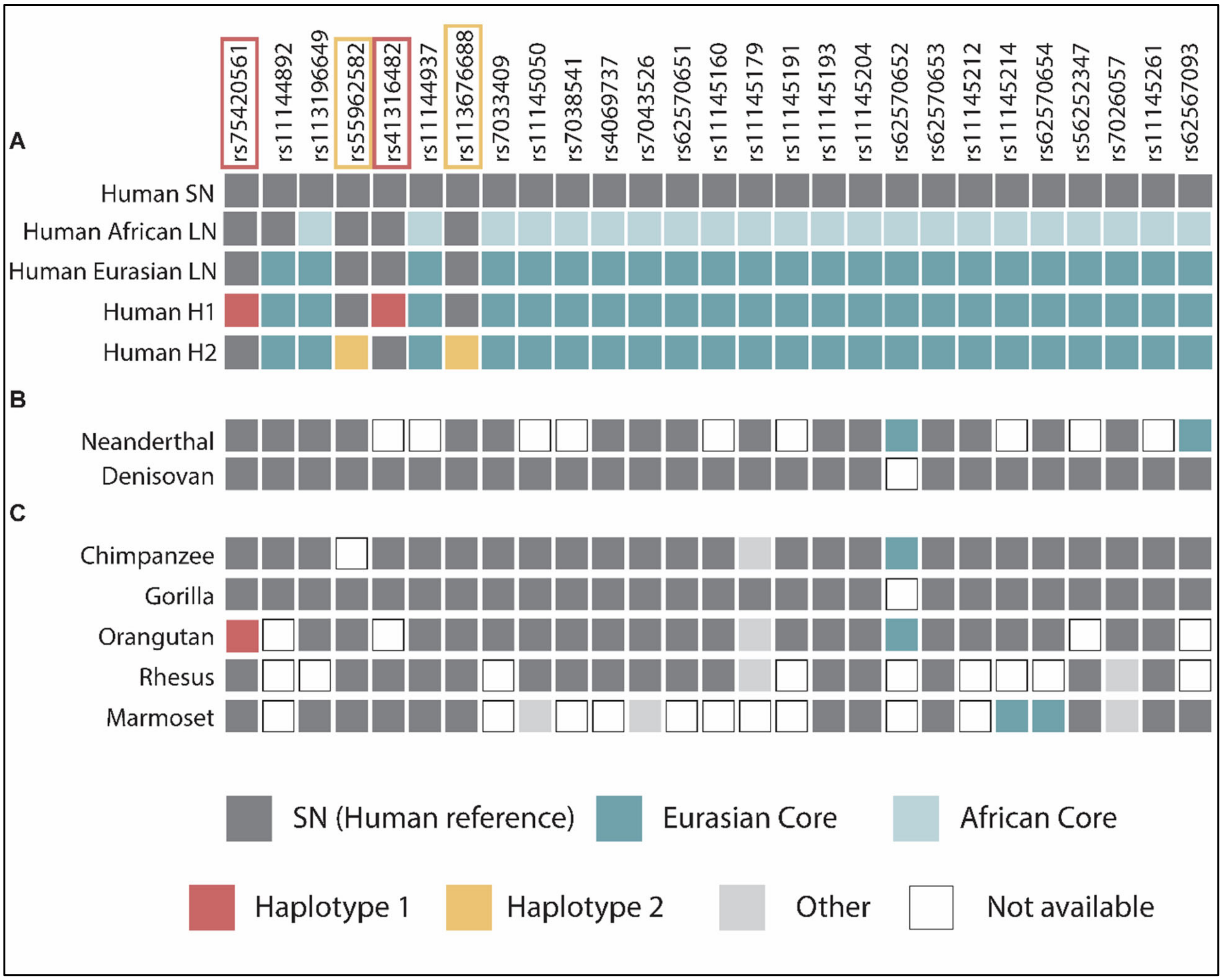

Supplementary Figure 4. The FRDA core-haplotype and major haplotypes (H1 and H2) of protomutation (PT) / E alleles are specific to modern humans. SNPs defining the FRDA core haplotype and the two major haplotypes of protomutation / E alleles (H1 & H2) are shown in: **(A)** Human alleles, including short normal (SN), long normal (LN; African and Eurasian haplotypes), and protomutation/expanded (PT/E) alleles (H1 and H2 haplotypes); **(B)** Archaic humans, including Neanderthal and Denisovan; and **(C)** Non-human primates, including chimpanzee, gorilla, orangutan, rhesus macaque (old world monkey), and marmoset (new world monkey). All 23 SNPs in the FRDA core haplotype (of which, 22 SNPs identify African LN alleles), plus the four additional SNPs in the H1 and H2 haplotypes, are ordered by genomic position (SNP IDs listed at the top), with each box indicating the observed allele for the corresponding sample or species. Color coding denotes the human reference allele (dark grey), human alternate alleles observed in LN (African [light blue] & Eurasian [dark blue]), or PT/E haplotypes (H1 [red] & H2 [yellow]), other alleles [light grey], or positions with no available data [white], as indicated in the key.

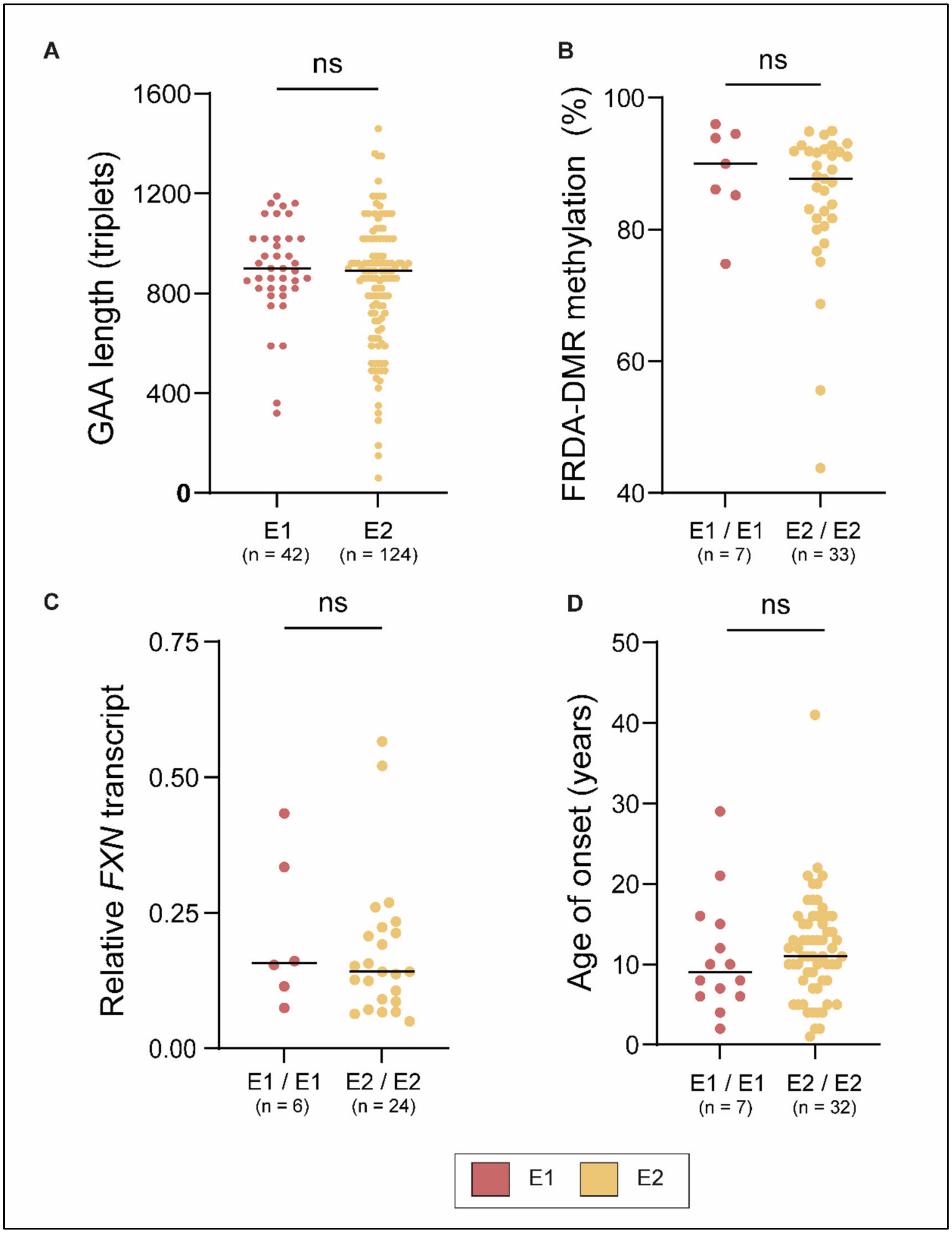

Supplementary Figure 5. Clinical and molecular features associated with the two major E allele haplotypes (E1 & E2). (A) Length of GAA repeat (triplets); note: each data point represents an individual allele. **(B)** DNA methylation (%) in the FRDA-DMR; **(C)** Relative *FXN* transcript levels; **(D)** Age of onset (years). Note: in **B-D**, each datapoint represents an FRDA patient who is homozygous for the E1 or E2 haplotype. Horizontal lines indicate group medians. Sample sizes are shown below each group. ns = not significant (Mann–Whitney test).

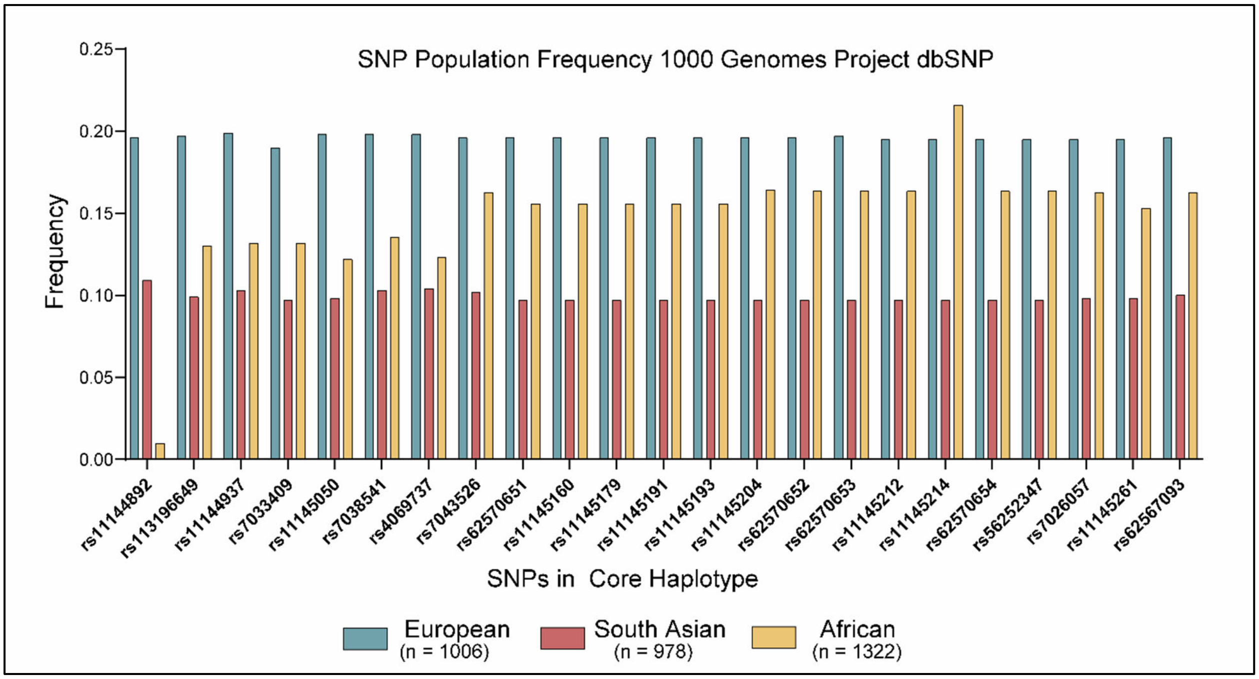

Supplementary Figure 6. SNP frequencies in the FRDA core haplotype in sub-Saharan Africa. Allele frequencies of SNPs included in the FRDA core haplotype (dbSNP; 1000 Genomes Project), in European, South Asian, and African populations.

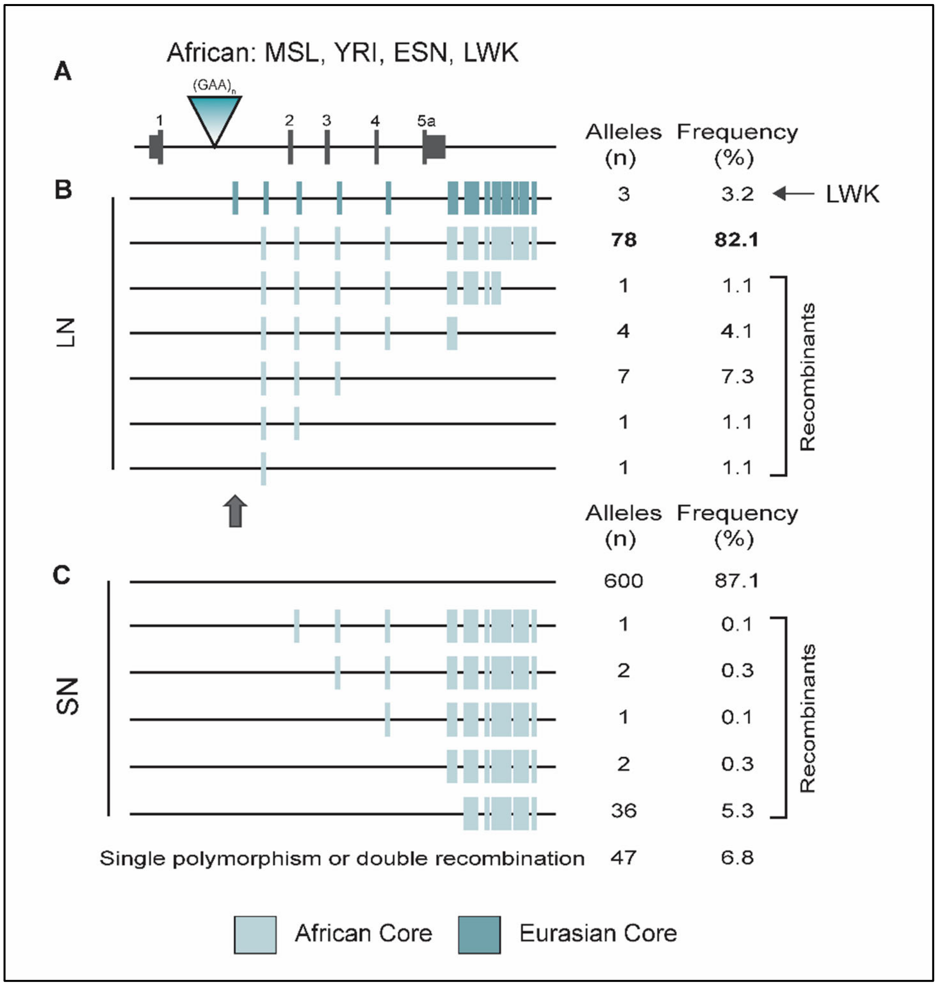

Supplementary Figure 7. Recombinant haplotypes in Sub-Saharan African populations. (A) Schematic of the *FXN* locus showing the GAA repeat in intron 1 and exons 1-5a. **(B)** The FRDA core haplotype, seen mostly in its entirety (82% show the 22 SNP core haplotype), or as 3' recombinants of the core haplotype (~15%), observed in LN alleles (n=95) in sub-Saharan African populations (MSL, YRI, ESN, LWK). Each horizontal line represents a distinct haplotype, with vertical marks indicating individual SNPs in the core haplotype. Allele counts (n) and frequencies (%) are indicated for all 95 LN alleles. Note that 3 alleles showed the complete 23 SNP core haplotype, including rs11144892 (vertical arrow), which is characteristic of Eurasians; all were from the LWK population (horizontal arrow). **(C)** Recombinant FRDA core SNPs observed among short normal (SN) alleles; i.e., partial African LN haplotypes in SN alleles. Haplotypes and SNPs are denoted as in panel B, with allele counts (n) and the frequencies (%) indicated of each haplotype among all SN alleles. Most SN alleles (87%) are non-recombinant. The rest are approximately evenly split between single recombinants, and other, more complex rearrangements.

Supplementary Tables

Population	Offspring			Mother			Father		
	ID	GAA length	Haplotypes	ID	GAA length	Haplotypes	ID	GAA length	Haplotypes
CEU	NA10840	9 / 30	SN / PT1	NA12287	20 / 30	PT 1 / PT2	NA12286		SN / SN
CEU	NA10859	9 / 20	SN / PT1	NA11882		SN / SN	NA11881	9 / 20	SN / PT1
CEU	NA10865	8 / 23	SN / PT2	NA11892		SN / SN	NA11891	9 / 22	SN / PT2
CEU	NA12485	8 / 26	SN / PT1	NA12414		SN / SN	NA12413	9 / 26	SN / PT1
CEU	NA12740	8 / 17	SN / PT1	NA12751		SN / SN	NA12750	8 / 17	SN / PT1
CEU	NA12817	9 / 20	SN / PT2	NA12828	8 / 20	SN / PT2	NA12827		SN / SN
CEU	NA12832	9 / 29	SN / PT2	NA12843	8 / 29	SN / PT2	NA12842		SN / LN
CEU	NA12865	6 / 22	SN / PT2	NA12875	18 / 22	LN / PT2	NA12874		SN / SN
IBS	HG01505	8 / 17	SN / PT1	HG01504	8 / 17	SN / PT1	HG01503		SN / SN
IBS	HG01532	9 / 17	SN / PT1	HG01531	8 / 17	SN / PT1	HG01530		SN / SN
IBS	HG01629	9 / 16	SN / PT1	HG01628	10 / 16	SN / PT1	HG01630		SN / SN
IBS	HG01763	9 / 25	SN / PT1	HG01761	9 / 25	SN / PT2	HG01762		SN / SN
IBS	HG01780	27 / 31	PT1 / PT2	HG01779	9 / 31	SN / PT2	HG01781	9 / 27	SN / PT2
IBS	HG02222	9 / 19	SN / PT1	HG02220	18 / 19	LN / PT1	HG02221		SN / LN
IBS	HG02240	5 / 25	SN / PT2	HG02239	8 / 25	SN / PT2	HG02238		SN / SN
PJL	HG03017	8 / 24	SN / PT1	HG03016	9 / 24	SN / PT1	HG03015		SN / SN

Supplementary Table 1. Intergenerational transmission of protomutation (PT) alleles in FRDA susceptible populations. Protomutation transmissions (both haplotypes; i.e., PT1 and PT2) were searched in all FRDA susceptible Eurasian populations available as trios from the Expanded 1000 Genomes Project. All 16 parent-child duos (accounting for 17 intergenerational transmissions) that were identified using available genomic sequences (which happened to be from CEU, IBS, and PJL populations) are listed. Genomic DNA samples were procured from the program for all 16 parent-child duos, and the GAA repeats were sequenced by longread amplicon sequencing. For each family, the offspring, and both parents are listed (with individual IDs), along with haplotype classifications. Repeat lengths are only reported for all parent-child duos showing intergenerational transmission of protomutation alleles. The single instance of intergenerational instability is highlighted in green. Population codes are as defined in the 1000 Genomes Project.

Sample ID	Genotype	Age	Location (Region)	Publication	Ancestry
**SF12**	PT2	8895	Sweden (Northern Europe)	1	Scandinavian hunter-gatherer
**AKT16**	PT1	8563	Turkey (East Europe)	2	Early Neolithic farmer with hunter-gatherer admixture
**LEPE52**	PT2	8093	Serbia (Southeast Europe)	2	Early Neolithic farmer
**STAR1**	PT2	7538	Serbia (Southeast Europe)	2	Early Neolithic farmer
**NE1**	PT1	7146	Hungary (Central Europe)	3	Early Neolithic farmer
**Iceman (Ötzi)**	PT1	5199	Italy (Southern Europe)	4	Early Neolithic farmer
**RISE150**	PT1	3744	Poland (Central Europe)	5	Unetice
**MA2203**	PT2	3575	Turkey (East Europe)	6	Hittite
**DA13**	PT2	2568	Kazakhstan (Central Asia)	7	Central Saka / Eastern Scythian
**VK522**	PT2	1550	Sweden (Northern Europe)	8	Viking
**VK491**	PT2	1200	Estonia (Northern Europe)	8	Viking
**DA243**	PT1	1050	Russia (Eastern Europe)	7	Caucasus Alan*
**VK166**	PT2	1010	United Kingdom (Northwest Europe	8	Viking
**VK266**	PT2	1000	Sweden (Northern Europe)	8	Viking
**VK279**	PT1	1000	Denmark (Northern Europe)	8	Viking
**VK446**	PT1	1000	Denmark (Northern Europe)	8	Viking
**VK129**	PT1	1000	Iceland (Northern Europe)	8	Viking
**VK345**	PT1	1000	Sweden (Northern Europe)	8	Viking
**VK479**	PT1	975	Sweden (Northern Europe)	8	Viking
**VK403**	PT1	900	Sweden (Northern Europe)	8	Viking
**VK160**	PT1	850	Russia (Eastern Europe)	8	Viking
**VK537**	PT1	800	Italy (Southern Europe)	8	Viking
**VK541**	PT1	700	Ukraine (Eastern Europe)	8	Viking

Supplementary Table 2. Ancient humans carrying one of two major haplotypes of protomutation (PT) and E alleles. Individuals are listed with their assigned haplotype (PT1 or PT2), age (years before present [1950 CE]), geographic location (modern-day location), source publication, and reported ancestry. Ancestry designations follow the archaeological and/or genetic classifications described in the original studies. Individuals above the dashed line are depicted in Fig. 6

**Alan individual from the North Caucasus, an Iranic-associated population.*

References:

1. Günther, T., Malmström, H., Svensson, E.M., Omrak, A., Sánchez-Quinto, F., Kılınç, G.M., Krzewińska, M., Eriksson, G., Fraser, M., Edlund, H., *et al.* (2018) Population genomics of Mesolithic Scandinavia: Investigating early postglacial migration routes and high-latitude adaptation. *PLOS Biol.*, **16**, e2003703.

2. Marchi, N., Winkelbach, L., Schulz, I., Brami, M., Hofmanová, Z., Blöcher, J., Reyna-Blanco, C.S., Diekmann, Y., Thiéry, A., Kapopoulou, A., *et al.* (2022) The genomic origins of the world's first farmers. *Cell*, **185**, 1842-1859.e18.
3. Gamba, C., Jones, E.R., Teasdale, M.D., McLaughlin, R.L., Gonzalez-Fortes, G., Mattiangeli, V., Domboróczki, L., Kővári, I., Pap, I., Anders, A., *et al.* (2014) Genome flux and stasis in a five millennium transect of European prehistory. *Nat. Commun.*, **5**, 5257.
4. Keller, A., Graefen, A., Ball, M., Matzas, M., Boisguerin, V., Maixner, F., Leidinger, P., Backes, C., Khairat, R., Forster, M., *et al.* (2012) New insights into the Tyrolean Iceman's origin and phenotype as inferred by whole-genome sequencing. *Nat. Commun.*, **3**, 698.
5. Allentoft, M.E., Sikora, M., Sjögren, K.-G., Rasmussen, S., Rasmussen, M., Stenderup, J., Damgaard, P.B., Schroeder, H., Ahlström, T., Vinner, L., *et al.* (2015) Population genomics of Bronze Age Eurasia. *Nature*, **522**, 167–172.
6. Damgaard, P., Martiniano, R., Kamm, J., Moreno-Mayar, J.V., Kroonen, G., Peyrot, M., Barjamovic, G., Rasmussen, S., Zacho, C., Baimukhanov, N., *et al.* (2018) The first horse herders and the impact of early Bronze Age steppe expansions into Asia. *Science*, **360**, eaar7711.
7. Damgaard, P. de B., Marchi, N., Rasmussen, S., Peyrot, M., Renaud, G., Korneliussen, T., Moreno-Mayar, J.V., Pedersen, M.W., Goldberg, A., Usmanova, E., *et al.* (2018) 137 ancient human genomes from across the Eurasian steppes. *Nature*, **557**, 369–374.
8. Margaryan, A., Lawson, D.J., Sikora, M., Racimo, F., Rasmussen, S., Moltke, I., Cassidy, L.M., Jørsboe, E., Ingason, A., Pedersen, M.W., *et al.* (2020) Population genomics of the Viking world. *Nature*, **585**, 390–396.

ID	Short-read whole genome sequencing			Longread amplicon sequencing	
	Depth of GAA coverage	ExpansionHunter	HipSTR	Depth of GAA coverage	Trgt
HG02239	36	8 / 25	8 / 8	5643	8 / 25
HG02224	44	8 / 17	8 / 17	1467	8 / 17
HG02223	35	9 / 18	9 / 9	6231	9 / 18
HG02221	34	9 / 20	9 / 19	5496	9 / 19
HG02220	42	18 / 20	18 / 20	1098	18 / 19
HG01781	42	9 / 27	9 / 9	1648	9 / 27
HG01779	48	9 / 31	9 / 9	1482	9 / 31
HG01773	40	9 / 15	9 / 15	8689	9 / 14
HG01761	67	9 / 22	9 / 9	959	9 / 25
HG01756	42	8 / 19	8 / 8	3347	8 / 21
HG01747	36	8 / 19	8 / 8	5655	8 / 17
HG01710	44	9 / 19	9 / 19	2206	9 / 19
HG01705	36	8 / 19	8 / 8	2781	8 / 15
HG01704	40	8 / 15	8 / 17	2545	8 / 17
HG01700	37	9 / 20	9 / 9	1846	9 / 19
HG01697	38	9 / 17	9 / 17	3801	9 / 17
HG01694	40	15 / 18	15 / 17	5346	17 / 18
HG01685	40	9 / 21	9 / 9	4482	9 / 20
HG01684	36	9 / 24	9 / 9	1542	9 / 23
HG01673	37	9 / 15	9 / 15	3215	9 / 15
HG01632	36	8 / 22	8 / 8	2726	8 / 22
HG01631	42	9 / 24	9 / 9	1355	9 / 23
HG01628	37	10 / 16	10 / 16	2017	10 / 16
HG01626	38	8 / 26	8 / 8	1690	8 / 24
HG01620	33	9 / 17	9 / 9	815	9 / 18
HG01618	37	9 / 24	8 / 9	3636	9 / 24
HG01613	42	9 / 19	9 / 19	3891	9 / 19
HG01612	43	9 / 26	9 / 9	7335	9 / 25
HG01610	39	8 / 19	8 / 8	2224	8 / 19
HG01531	40	8 / 15	8 / 17	3198	8 / 17
HG01527	41	19 / 20	19 / 20	1605	18 / 19
HG01504	39	8 / 18	8 / 8	2118	8 / 17

Supplementary Table 3. GAA repeat lengths measured by longread amplicon sequencing is superior to bioinformatic estimates (ExpansionHunter or HipSTR) derived from short-read whole genome sequences. All IBS individuals who were positive (i.e., at least one allele) for the FRDA core haplotype were selected as a test cohort for side-by-side comparison of both methods. Repeat lengths were estimated from short-read whole genome sequencing (1000 Genomes Project) using ExpansionHunter and HipSTR. Additionally, long-read amplicon sequencing was performed to directly measure the consensus GAA repeat length using Trgt. Depth GAA repeat coverage and estimated repeat lengths are shown for each method. Short-read whole genome sequencing yielded an average of 40X reads at the GAA repeat, and longread amplicon sequencing produced an average of 3190X reads. Instances where discrepancies were identified versus high-confidence reads from longread amplicon sequencing are indicated in red and instances where HipSTR measured only the SN allele are indicated in blue.

Supplementary Data

Supplementary Data 1: See <u>Devore et al. Supplementary Data 1.xlsx</u>

Nextgen sequencing and SNP identification in FRDA and non-FRDA controls.

Supplementary Data 2: See <u>Devore et al. Supplementary Data 2.xlsx</u>

GAA repeat sequences and *FXN* haplotypes for individuals from the 1000 Genomes Project who have at least one allele with the FRDA core haplotype (European, South Asian, and African: IBS, GBR, TSI, PJL, GIH, ITU, ESN, YRI, MSL, LWK).

Supplementary Data 3: See <u>Devore et al. Supplementary Data 3.xlsx</u>

Ancient DNAs with informative coverage at *FXN* haplotype-defining SNPs.
